# The Nedd8‐activating enzyme inhibitor MLN4924 (TAK‐924/Pevonedistat) induces apoptosis via c‐Myc‐Noxa axis in head and neck squamous cell carcinoma

**DOI:** 10.1111/cpr.12536

**Published:** 2018-10-19

**Authors:** Wenjuan Zhang, Yupei Liang, Lihui Li, Xiaofang Wang, Zi Yan, Changsheng Dong, Mu‐Sheng Zeng, Qian Zhong, Xue‐Kui Liu, Jinha Yu, Shuyang Sun, Xiaojun Liu, Jihui Kang, Hu Zhao, Lak Shin Jeong, Yanmei Zhang, Lijun Jia

**Affiliations:** ^1^ Cancer Institute Fudan University Shanghai Cancer Center Shanghai China; ^2^ Cancer Institute Longhua Hospital Shanghai University of Traditional Chinese Medicine Shanghai China; ^3^ Department of Experimental Research Sun Yat‐sen University Cancer Center State Key Laboratory of Oncology in South China Collaborative Innovation Center for Cancer Medicine Guangzhou China; ^4^ Department of Head & Neck Cancer Sun Yat‐sen University Cancer Center State Key Laboratory of Oncology in South China Collaborative Innovation Center for Cancer Medicine Guangzhou China; ^5^ College of Pharmacy Seoul National University Seoul Korea; ^6^ Department of Oral and Maxillofacial‐Head Neck Oncology Ninth People's Hospital Shanghai Jiao Tong University School of Medicine Shanghai China; ^7^ Department of Clinical Laboratory Huadong Hospital Shanghai Key Laboratory of Clinical Geriatric Medicine Research Center on Aging and Medicine Fudan University Shanghai China

## Abstract

**Objectives:**

The present study aimed to reveal expression status of the neddylation enzymes in HNSCC and to elucidate the anticancer efficacy and the underlying mechanisms of inhibiting neddylation pathway.

**Materials and methods:**

The expression levels of neddylation enzymes were estimated by Western blotting in human HNSCC specimens and bioinformatics analysis of the cancer genome atlas (TCGA) database. Cell apoptosis was evaluated by Annexin V fluorescein isothiocyanate/propidium iodide (Annexin V‐FITC/PI) stain and fluorescence‐activated cell sorting (FACS). Small interfering RNA (siRNA) and the CRISPR‐Cas9 system were used to elucidate the underlying molecular mechanism of MLN4924‐induced HNSCC apoptosis.

**Results:**

Expression levels of NAE1 and UBC12 were prominently higher in HNSCC tissues than that in normal tissues. Inactivation of the neddylation pathway significantly inhibited malignant phenotypes of HNSCC cells. Mechanistic studies revealed that MLN4924 induced the accumulation of CRL ligase substrate c‐Myc that transcriptionally activated pro‐apoptotic protein Noxa, which triggered apoptosis in HNSCC.

**Conclusions:**

These findings determined the over‐expression levels of neddylation enzymes in HNSCC and revealed novel mechanisms underlying neddylation inhibition induced growth suppression in HNSCC cells, which provided preclinical evidence for further clinical evaluation of neddylation inhibitors (eg, MLN4924) for the treatment of HNSCC.

## 1 INTRODUCTION

Protein neddylation is a type of posttranslational modification, which conjugates neural precursor cell expressed, developmentally down‐regulated 8 (NEDD8), a ubiquitin‐like molecule, to targeted proteins and then affects subcellular localization, stability, conformation and function of targeted proteins.^1^, ^2^, ^3^, ^4^, ^5^ This process is a three‐step enzymatic cascade involving NEDD8‐activating enzyme E1 (NAE, a heterodimer comprising subunits NAE1 and UBA3), NEDD8‐conjugating enzyme E2 M (UBC12) and substrate‐specific E3s.^1^, ^2^, ^3^, ^4^, ^5^ Cullin family proteins, which serve as essential components of cullin‐RING E3 ubiquitin ligases (CRLs), are the best‐known substrates among NEDD8‐targeted proteins.^6^, ^7^ Modification of cullin by NEDD8 leads to the activation of CRL, which further triggers the ubiquitination and degradation of its substrates to regulate diverse biological processes, such as transcription, signal transduction, cell‐cycle progression and stress responses. The dysfunction of CRL, such as the elevated CRL neddylation modification, contributes to carcinogenesis and cancer progression.^8^ Recently, the neddylation pathway, including NAE, UBC12 and NEDD8 itself, has been frequently reported to be hyperactivated in several human cancers and indicates an unfavourable prognosis, highlighting the neddylation‐CRL pathway as an attractive anticancer target.^9^, ^10^, ^11^, ^12^, ^13^

MLN4924 (Pevonedistat /TAK‐924), an investigational small‐molecule inhibitor of NAE, has shown antitumor activity in various cancer xenograft models.^9^, ^11^, ^12^, ^14^, ^15^ Mechanistically, MLN4924 abrogates cullin neddylation, and therefore inactivates CRL, leading to the accumulation of tumour‐suppressive CRL substrates to suppress the growth of cancer cells by triggering cell‐cycle defects, apoptosis or senescence.^3^, ^9^, ^16^, ^17^, ^18^, ^19^ Preclinical studies have demonstrated the therapeutic efficacy of MLN4924 as a single anticancer agent^9^, ^11^, ^14^, ^15^ or in combination with chemo/radiotherapy.^20^, ^21^ Due to its potent anticancer efficacy and well‐tolerated toxicity in preclinical studies, MLN4924 is currently tested in several Phase I/II clinical trials for relapsed/refractory lymphoma, multiple myeloma and advanced nonhematologic malignancies (http://www.clinicaltrials.gov).^22^, ^23^, ^24^ Encouragingly, MLN4924 demonstrates anticipated pharmacodynamics effects in myelodysplastic syndromes (MDS), acute myeloid leukaemia (AML), lymphoma and solid tumours with a tolerable safety profile in recently published clinical trials.^22^, ^23^, ^24^

Head and neck squamous cell carcinoma (HNSCC) is the sixth most common cancer worldwide, with an incidence of ~600 000 cases per year and mortality of ~50%.^25^, ^26^ Despite advances in therapeutic approaches over the past decades, little improvement has been achieved in the survival rate for HNSCC due to relatively low anticancer efficacy, severe treatment‐associated adverse effect and acquired drug resistance, leading to high risk of local recurrences and the development of distant metastases.^27^, ^28^ This plight makes an urgent necessity to identify novel anticancer targets and develop new therapeutic agents with efficient and selective anticancer efficacy to improve the treatment of HNSCC.

A previous study has reported that highly proliferative HNSCC cells possessed up‐regulated NEDD8 conjugation and MLN4924 cooperating with TRAIL‐augmented apoptosis possibly through facilitating c‐FLIP degradation in HNSCC cells.^29^ Most recently, Vanderdys et al^30^ found that Pevonedistat suppressed and radiosensitized HNSCC through inactivating CRL4‐CDT2 and DNA re‐replication. Moreover, tumour biopsies of patients with head and neck cancer exhibited the elevated CRL substrates CDT1 and NRF2 after MLN4924 treatment, indicating MLN4924 as an effective neddylation inhibitor and a potent clinical strategy for the treatment of HNSCC.^23^ However, the underlying mechanisms of anti‐HNSCC effects of MLN4924 remain elusive. In this study, the anti‐HNSCC efficacy and its underlying mechanisms of MLN4924 have been elegantly defined. We found that neddylation inhibition by MLN4924 significantly suppressed HNSCC malignant phenotypes by inducing G_2_ phase cell cycle arrest and c‐Myc/Noxa axis‐dependent apoptosis.

## 2 MATERIALS AND METHODS

### 2.1 Cell lines, culture and reagents

HNSCC cell lines CAL27 (ATCC) and HN13^31^ were granted by Dr. Shuyang Sun (Department of Oral and Maxillofacial‐Head Neck Oncology, Ninth People's Hospital, Shanghai Jiao Tong University School of Medicine). Cells were routinely cultured in Dulbecco's modified Eagle's medium (Gibco, Thermo Fisher Scientific, Grand Island, NY, USA) with 10% FBS (Biochrom, Holliston, MA, USA) and 1% penicillin‐streptomycin solution, a humidified atmosphere of 5% CO_2_ and 95% air at 37°C. MLN4924 was synthesized and prepared as previously described.^9^

### 2.2 Cell viability assay and clonogenic survival assays

Cells were seeded in 96‐well plates (3 × 10^3^ cells/well) and treated with DMSO or MLN4924. Cell proliferation was determined using the ATPlite Luminescence Assay Kit (PerkinElmer, Waltham, MA, USA) according to manufacturer's protocol.^9^ For the clonogenic assay, 200 cells were seeded in 6‐well plates and then were treated with DMSO or MLN4924 and incubated for 10 days in 6‐well plates. Colonies comprising 50 cells or more were counted under an inverted microscope.^32^ Three independent experiments were performed.

### 2.3 Transwell cell migration and invasion assays

A standard transwell cell migration assay using a transwell polycarbonate filter (8 μm pore size; Corning Inc., Lowell, MA, USA) was performed to analyse the efficacy of MLN4924 on cell migration.^9^ Briefly, cells suspended with serum‐free DMEM (Gibco, Thermo Fisher Scientific, Grand Island, NY, USA) containing an indicated concentration of MLN4924 were adjusted to 6 × 10^5^ cells/mL for HN13 and 1 × 10^6^ cells/mL for CAL27. The suspension was added into the upper chambers, with 100 μL per chamber. DMEM, containing 10% foetal bovine serum (FBS), was added in the lower chambers, with 600 μL per chamber. The cells were cultured for 24 h. Cells that migrated to the underside of the upper chambers were stained with 0.1% crystal violet for 30 min. Cells that passed through the polycarbonate membrane of the wells were counted under a Leica microscope. The representative results of three independent experiments with similar trend were presented.

As for cell invasion assay, BD BioCoat™ Matrigel™ Invasion Chambers (8 μm pore size; BD Biosciences, San Diego, CA, USA) were applied, and the culture time was prolonged to 36 h.

### 2.4 Immunoblotting and cycloheximide (CHX)‐chase assay

Cell lysates were prepared and analysed by immunoblotting. Antibodies against P21 (AB109199) and UBC12 (AB116248) were purchased from Abcam (Shanghai, China). Antibody against Noxa (OP180) was from Millipore (Merck KGaA, Darmstadt, Hesse, Germany). Antibodies against NAE1 (HPA042041) were purchased from Sigma‐Aldrich (Merck KGaA, Darmstadt, Hesse, Germany). Antibodies against P27 (3686), WEE1 (4936), p‐histone 3(3377), cleaved‐Caspase‐3 (9661), cleaved‐Parp (5625), Caspase‐8 (9746), cleaved‐Caspase‐9 (9501), Bad (9239), Bid (2002), Bax (5023), Bak (6947), Bim (2933) and Bik (4592) were from Cell Signaling Technology (Danvers, MA, USA). For CHX‐chase experiments, cells were treated with 50 μg/mL CHX (Sigma, C4859, Merck KGaA, Darmstadt, Hesse, Germany) in combination with 1.0 μmol/Lol/L MLN4924 or DMSO for indicated time points^9^ .

### 2.5 Cell‐cycle profile analysis

Cell‐cycle profile was evaluated by propidium iodide (PI) staining and fluorescence‐activated cell sorting (FACS) analysis as described previously.^33^, ^34^ Briefly, cells were treated with MLN4924 or dimethyl sulfoxide (DMSO) then fixed with 70% ethanol at −20°C overnight, stained with PI (36 μg/mL; Sigma‐Aldrich, P4170, Merck KGaA, Darmstadt, Hesse, Germany) containing RNase A (10 μg/mL; Sigma‐Aldrich, R6513) at 37°C for 15 min and analysed for cell‐cycle profile by CyAn ADP (Beckman Coulter, Brea, CA, USA). Data were analysed with ModFit LT software (Verity Software House, Topsham, ME, USA).

### 2.6 Detection of apoptosis

Cells were treated with the indicated concentration of MLN4924 for 48 h. Apoptosis was determined with the Annexin V‐FITC/PI Apoptosis Kit (BD Biosciences, San Diego, CA, USA) according to manufacturer's instructions.^9^

### 2.7 Gene silencing using small interfering RNA (siRNA)

HN13 cells were transfected with siRNA oligonucleotides and synthesized by GenePharma (Shanghai, China) using Lipofectamine 2000. The sequences of the siRNA were as follows: siNoxa, 5′‐GUAAUUAUUGACACAUUUC‐3′^35^; siBim, 5′‐GACCGAGAAGGUAGACAAUUG‐3′^36^; siBik, 5′‐AAGACCCCUCUCCAGAGACAU‐3′^37^; sic‐Myc, 5′‐CGAGCUAAAACGGAGCUUU‐3′^38^; and siATF4, 5′‐GCCUAGGUCUCUUAGAUGA‐3′.^39^

### 2.8 RNA extraction and quantitative polymerase chain reaction (qPCR)

Total RNA was extracted using the Ultrapure RNA Kit (CWbiotech, Beijing, China). RNA (1.0 μg) was purified and reversely transcribed by PrimeScript^®^ RT Master (Takara, Dalian, China) following manufacturer's instructions. The cDNA was quantified by real‐time qPCR using SYBR^®^ Green Real‐Time PCR Master Mixes (Applied Biosystems, Foster City, CA, USA) and a real‐time PCR system (Applied Biosystems, Thermo Fisher Scientific, Grand Island, NY, USA) according to manufacturer's instructions.^9^ For each sample, the mRNA abundance was normalized to the amount of actin. Primers were as follows. Noxa: forward, 5′‐GAGATGCCTGGGAAGAAGG‐3′; reverse, 5′‐TTCTGCCGGAAGTTCAGTTT‐3′. Bik: forward, 5′‐TTCATCTACGACCAGACT‐3′; reverse, 5′‐ATCTCCAGAACCTCATTATG‐3′. Bim: forward, 5′‐GCAGATATGCGCCCAGAGAT‐3′; reverse, 5′‐AAGCGTTAAACTCGTCTCCGATA‐3′. Actin: forward, 5′‐TGACGTGGACATCCGCAAAG‐3′; reverse, 5′‐CTGGAAGGTGGACAGCGAGG‐3′.

### 2.9 Collection of HNSCC tissues and clinicopathological characteristics of patients

All patients underwent surgery, followed by treatment in accordance with the National Comprehensive Cancer Network Clinical Practice Guidelines. Fresh HNSCC tissues with paired adjacent normal tissues (with more than a 5 mm distance from the primary tumour's edge) of 20 HNSCC patients were collected at the time of operation between 2014 and 2015 at the Sun Yat‐sen University Cancer Center. Written informed consents regarding tissue and data used for scientific purposes were obtained from all participating patients. The study was approved by the Research Ethics Committee of Sun Yat‐sen University Cancer Center.

### 2.10 Generation of stable cell lines

Knockout was performed by SgRNA oligos (Table S1) into lentiCRISPR v2 plasmid (Plasmid #52961, Addgene, Cambridge, MA, USA). LentiCRISPR plasmid with SgRNA (4.0 ng), packaging plasmids psPAX2 (3.0 ng) and pMD2.G (1.0 ng) were transfected into HEK293T cells in 5 mL Opti‐MEM medium with Lipofectamine 2000, and virus supernatant was harvested 36 h post‐transfection and mixed with polybrene to increase infection efficiency. The infected HN13 cells were selected with 2 μg/mL puromycin for two weeks.^40^

### 2.11 Bioinformatics analysis

TCGA RNA‐Seq and corresponding clinical data were based upon data generated by TCGA Research Network (https://cancergenome.nih.gov/). RNA‐Seq analysis was performed for the data from 514 HNSCC tissues and 74 adjacent normal tissues.

### 2.12 Statistical analysis

Survival was analysed using the Kaplan‐Meier method and compared using the log‐rank test with Statistical Program for Social Sciences software (SPSS) version 17.0. The overall survival time was defined as the duration from the date of diagnosis to the date of either death or censoring (which could occur either by loss to follow‐up or by termination of the observation).

The statistical significance of differences between groups was assessed using GraphPad Prism5 software (GraphPad Software, San Diego, CA, USA). The *t* test was used for the comparison of parameters between groups. Data are presented as mean ± standard deviation. For all tests, three levels of significance (*P <* *0.05, **P <* *0.01, ***P <* *0.001) were applied^9^

## 3 RESULTS

### 3.1 Inhibiting neddylation pathway impeded the maintenance of HNSCC malignant phenotypes

**Figure 1:**
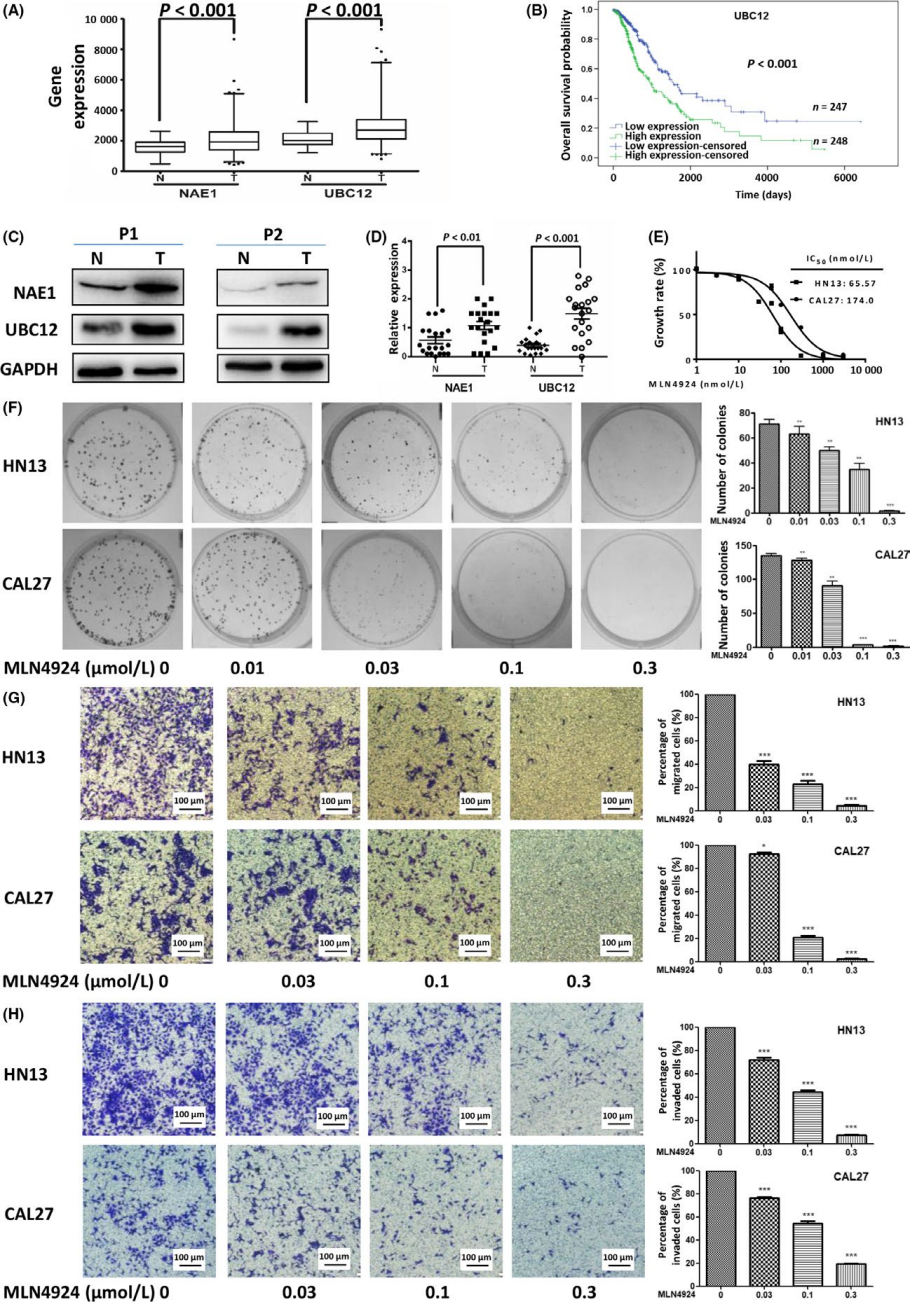
Inhibiting neddylation pathway impeded the maintenance of HNSCC malignant phenotypes. (A) Bioinformatics analysis of TCGA RNA‐Seq database. Gene expression levels of NAE1 and UBC12 were higher in HNSCC tissues (n = 514) than adjacent normal tissues (n = 74). (B) High expression of UBC12 indicated an unfavourable prognosis. Kaplan‐Meier curves for overall survival probability of patients with HNSCC according to the expression of UBC12 with median gene expression level as the cut‐off value (*P* < 0.001, log‐rank test). (C, D) Expression levels of NAE1 and UBC12 in clinical HNSCC tissues and adjacent normal tissues. Representative results of two (P1 and P2) of 20 pairs of tissues were determined by immunoblot (C). Quantification of protein expression levels in 20 pairs of HNSCC tumour tissues compared with adjacent normal tissues was analysed (D). (E) Growth suppression of HNSCC cells induced by MLN4924. 3 × 10^3^ HN13 and CAL27 cells were seeded in 96‐well plates in triplicates and treated with various concentrations of MLN4924 for 72 h and then lysed for ATPlite assay (mean ± SD, n = 3). (F) MLN4924 suppressed colony formation in HNSCC cells. HN13 and CAL27 cells were seeded into 6‐well plates with 200 cells per dish and cultured overnight and then treated with indicated doses of MLN4924 for 10 days, followed by crystal violet staining and colony counting (***P < 0.001, n = 3). (G, H) MLN4924 suppressed cell migration and invasion in HNSCC cells. HN13 and CAL27 cells were treated with MLN4924 at indicated doses to determine its therapeutic efficacy on cell migration (G; ***P < 0.001, n = 3) and invasion (H; ***P < 0. 001, n = 3). These data were representative results of three independent experiments with similar trends. Data represented means, and error bars were standard deviation. Two‐sided *t* test. N = adjacent normal tissues. T = tumour tissues. IC50 = half‐maximal inhibitory concentrations

To determine the expression status of the neddylation enzymes in HNSCC, bioinformatics analysis of TCGA RNA‐Seq database was performed to determine the expression levels of NEDD8‐activating enzyme E1 (*NAE1*) and NEDD8‐conjugating enzyme E2 (*UBC12*). As shown in Figure 1A, the mRNA levels of *NAE1* and *UBC12* in TCGA RNA‐Seq database detected HNSCC tissues (n* *=* *514) were significantly higher than those in detected adjacent normal tissues (n* *=* *74) (both *P *<* *0.001). Moreover, Kaplan‐Meier analysis showed that the overall survival probability was lower in HNSCC patients with high expression of UBC12 than in patients with low expression of UBC12 (*P *<* *0.001) (Figure 1B). To validate bioinformatics analysis results of TCGA RNA‐Seq, immunoblotting of 20 pairs of clinical samples with HNSCC tissues and adjacent normal tissues was further performed to determine the protein levels of NAE1 and UBC12. Consistent with up‐regulated gene expression levels, the protein levels of NAE1 (60%, 12/20) and UBC12 (70%, 14/20) were higher in HNSCC tissues than in adjacent normal tissues (Figure 1C,D). These data collectively revealed an over‐activated status of the neddylation pathway in HNSCC.

After validating the up‐regulated status of the neddylation enzymes in HNSCC, we evaluated the anticancer effect of inhibiting neddylation pathway in HNSCC cells with MLN4924. First, two HNSCC cell lines, CAL27 and HN13, were treated with MLN4924 for 72 h, and the half‐maximal inhibitory concentrations (IC_50_) were determined as 174.0 nmol/L (CAL27) and 65.57 nmol/L (HN13) (Figure 1E), suggesting that MLN4924 is an effective anti‐cancer agent in HNSCC cells. Meanwhile, the colony formation assay showed that MLN4924 inhibited clonogenic cell survival in CAL27 and HN13 cells in a dose‐dependent manner (Figure 1F). Furthermore, MLN4924 exerted a dose‐dependent inhibition of transwell migration and invasion assay on CAL27 and HN13 cell lines (Figure 1G,H). These findings demonstrated the anti‐HNSCC effects of neddylation inhibition by MLN4924.

### 3.2 MLN4924 triggered G_2_ phase cell‐cycle arrest by blocking the degradation of P27, P21 and WEE1

**Figure 2:**
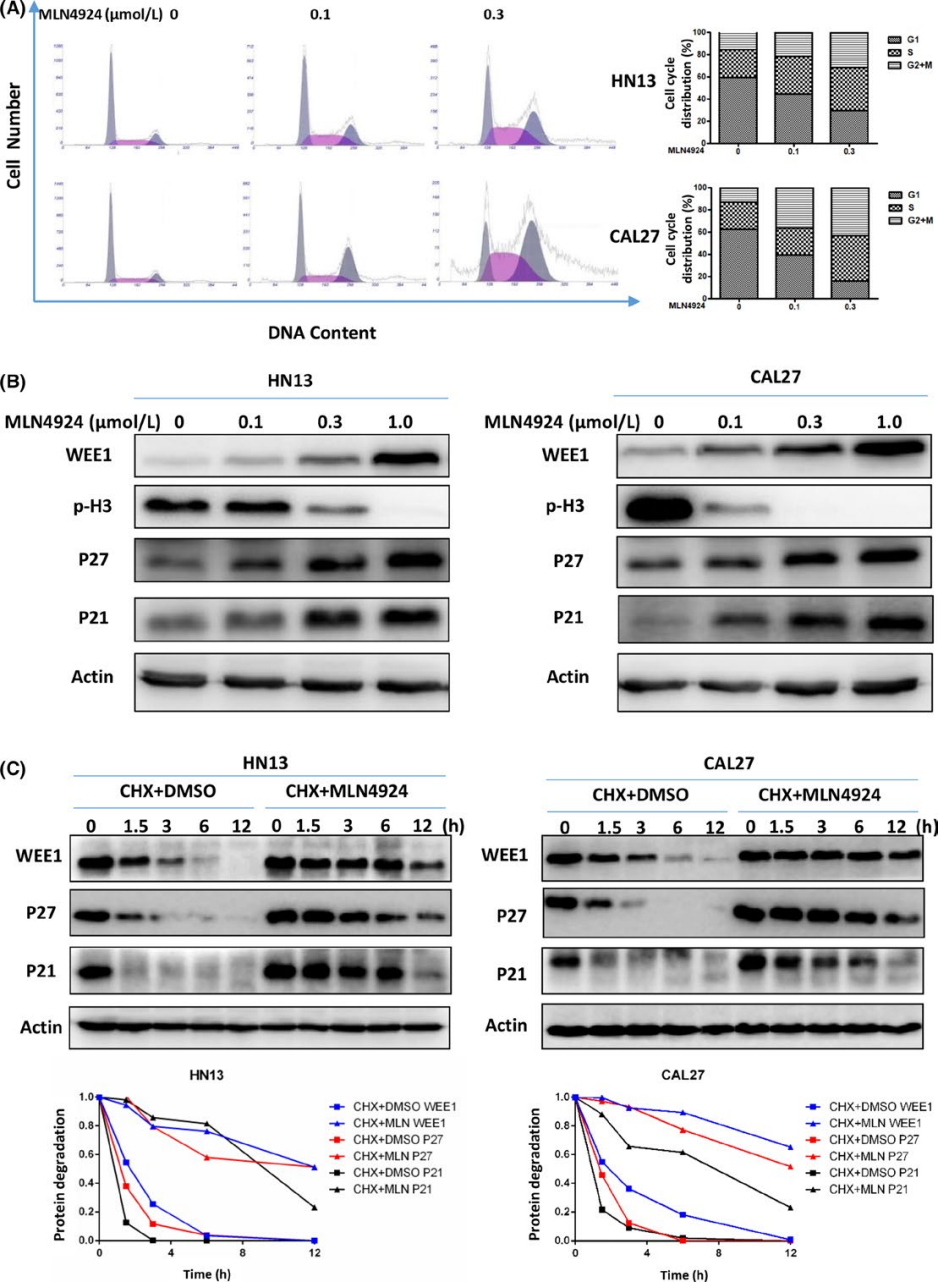
Neddylation inhibition with MLN4924 triggers G2 cell‐cycle arrest. (A) MLN4924‐induced G2‐M cell‐cycle arrest. HN13 and CAL27 cells seeded into 60‐mm dishes were treated with MLN4924 at 0.1 and 0.3 μmol/L vs DMSO for 24 h, followed by PI staining and FACS analysis for cell‐cycle profile. (B) MLN4924‐induced accumulation of P21, P27 and WEE1 accompanied by the decrease in p‐H3. Twenty‐four hour after HNSCC cells treated with MLN4924 at increasing concentrations (0.1, 0.3 and 1.0 μmol/L) vs DMSO, cells were subjected to immunoblotting using antibodies against WEE1, p‐H3, P27 and P21 with actin as a loading control. (C) MLN4924 prolonged the half‐lives of WEE1, P27 and P21. HN13 and CAL27 cells were treated with 1.0 μmol/L MLN4924 vs DMSO in combination with 50 μg/mL CHX for 0, 1.5, 3, 6 and 12 h, and then subjected to immunoblotting using antibodies against WEE1, P27 and P21 with actin as a loading control. The protein level was quantified by densitometric analysis

To elucidate the underlying mechanisms of MLN4924‐induced inhibitory effects on HNSCC cells, cell‐cycle profiles of MLN4924‐treated cells were analysed by using PI staining and FACS analysis after 24‐h treatment. As shown in Figure 2A, a prominent G_2_‐M phase cell‐cycle arrest was observed in both CAL27 and HN13 cells in a dose‐dependent manner. The expression of WEE1, an inhibitor of G_2_‐M phase transition,^41^ and Phospho‐Histone H3 (p‐H3), a hallmark of M phase cells,^42^ was further detected after MLN4924 treatment. As shown in Figure 2B, MLN4924 induced the accumulation of WEE1 and decrease in p‐H3, indicating that MLN4924‐treated cells were arrested at the G_2_ phase.

To address the potential mechanisms of MLN4924‐induced G_2_ phase arrest, the expression levels of cell cycle‐inhibitory CRL substrates, P21 and P27, were determined upon MLN4924 treatment.^43^, ^44^ As shown in Figure 2B, MLN4924 led to the accumulation of both P27 and P21. Considering that P27, P21 and WEE1 serve as the substrates of CRL E3 ligase, we hypothesized that MLN4924 induced the accumulation of these cell cycle‐related proteins by blocking their degradation due to CRL inactivation. To test this hypothesis, the degradation of P27, P21 and WEE1 in MLN4924‐treated cells was determined under the condition of protein translation inhibition by CHX. As shown in Figure 2C, neddylation inhibition significantly stabilized the expression of these cell cycle‐related proteins and extended their half‐lives in MLN4924‐treated cells.

### 3.3 MLN4924‐activated apoptotic pathway in HNSCC cells

**Figure 3:**
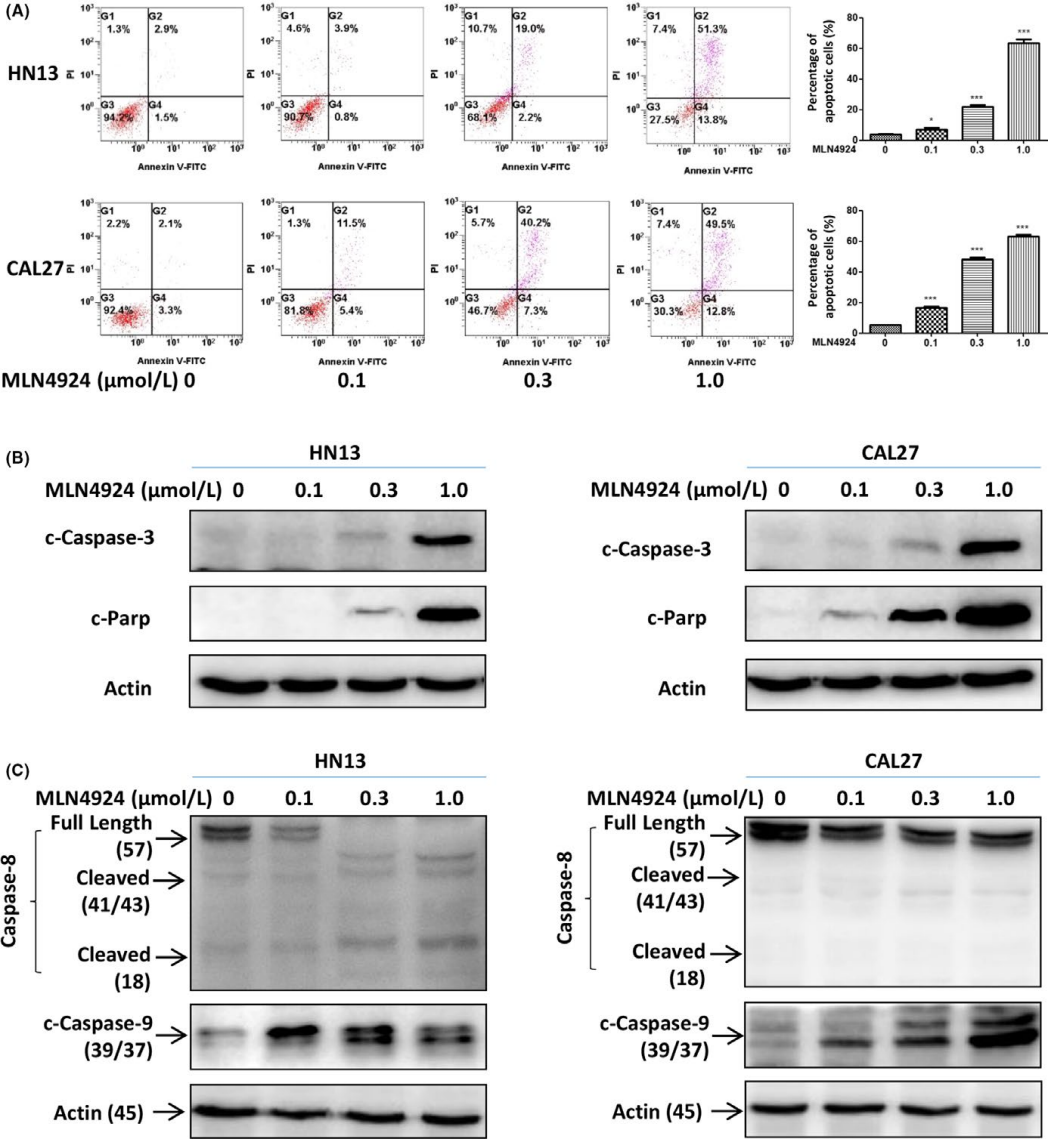
Treatment upon MLN4924 led to the activation of apoptotic pathway in HNSCC cells. (A, B) MLN4924‐induced apoptosis in a dose‐dependent manner. HN13 and CAL27 cells, treated with MLN4924 at increasing concentrations (0.1, 0.3 and 1.0 μmol/L) vs DMSO for 48 h, were subjected to Annexin V‐FITC/PI double‐staining analysis (A) and to immunoblotting using antibodies against cleaved‐Caspase‐3 (c‐Caspase‐3) and cleaved‐Parp (c‐Parp) (B) (*P < 0.05,***P < 0.001, n = 3). (C) Intrinsic apoptosis pathway was mainly activated upon neddylation inhibition. HN13 and CAL27 cells were treated by the way mentioned above and were subjected to immunoblotting using antibodies against Caspase‐8, cleaved‐Caspase‐9 (c‐Caspase‐9), with actin as a loading control. These data were representative of three independent experiments. Data represented means, and error bars were standard deviation. Two‐sided *t* test

To further define the cellular response to neddylation inhibition over time, HNSCC cells were exposed to MLN4924 with prolonged time (48 h) and observed under microscope. Interestingly, both treated HN13 and CAL27 cells presented a notable feature of apoptosis‐shrunk morphology in shape (Figure S1A), indicating that long‐term (48 h) exposure of cells to MLN4924 led to apoptosis. Consistently, MLN4924 treatment significantly increased the Annexin V‐positive cell population in both treated cell lines (Figure 3A). The induction of apoptosis was further validated by the appearance of cleaved‐Caspase‐3 and cleaved‐Parp, two classical hallmarks of apoptosis (Figure 3B).

To define MLN4924‐activated apoptotic pathways, the cleavage of Caspase‐8 and Caspase‐9, two representative hallmarks of extrinsic and intrinsic apoptotic pathways, respectively, was determined after treatment. As shown in Figure 3C, the cleavage of Caspase‐9 was significantly induced by MLN4924 in both cell lines, whereas the cleaved‐Caspase‐8 was only detected in HN13 cell line, indicating that the intrinsic apoptotic pathway was mainly activated upon neddylation inhibition.

### 3.4 MLN4924 triggered apoptosis via the up‐regulation of BH3‐only protein Noxa

**Figure 4:**
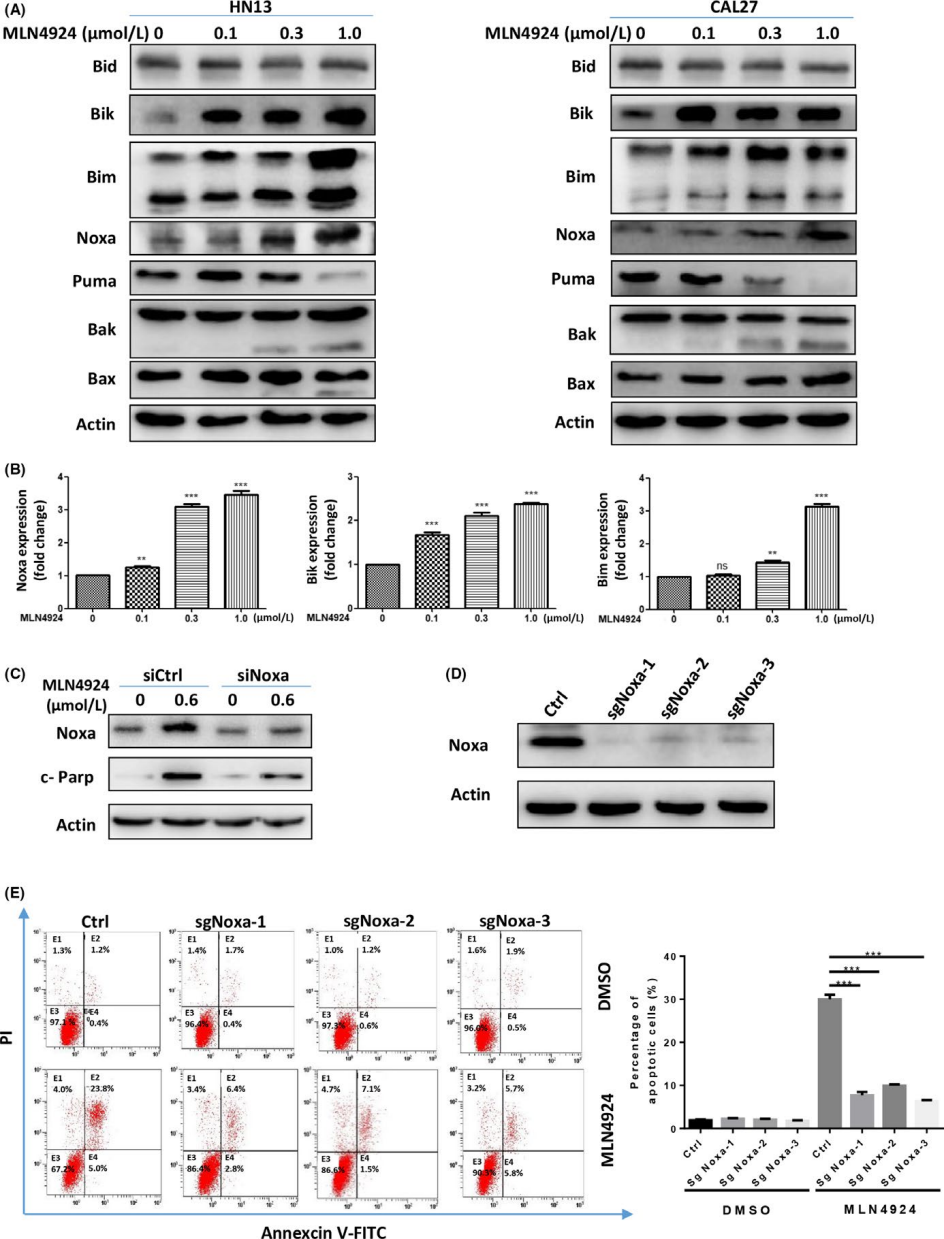
MLN4924 triggered apoptosis via the up‐regulation of BH3‐only proteins. (A) Effects of MLN4924 on the expression levels of pro‐apoptotic proteins. HN13 and CAL27 cells were treated with MLN4924 at increasing concentrations (0.1, 0.3 and 1.0 μmol/L) vs DMSO for 48 h, followed by immunoblotting using indicated antibodies against pro‐apoptotic proteins with Actin as a loading control. (B) Effects of MLN4924 on transcriptional activation of Noxa, Bim and Bik. HN13 cells were treated by the way mentioned above and were subjected to the real‐time PCR analysis (**P < 0.01, ***P < 0.001, n = 3). (C) Knockdown of Noxa attenuated the apoptosis induced by MLN4924. After HN13 cells transfected Noxa vs control siRNA for 48 h, cultures were treated with DMSO or 0.6 μmol/L MLN4924 for another 48 h. Apoptosis induction was quantified by immunoblotting using antibody against cleaved‐Parp (c‐Parp) with Actin as a loading control. (D, E) Knockout of Noxa attenuated the apoptosis induced by MLN4924. (D) Noxa knockout efficiency was determined by Western blotting with the indicated antibodies with actin as a loading control. (E) Noxa knockout cell lines were treated with DMSO or 0.6 μmol/L MLN4924 for 48 h. Apoptosis induction was quantified by Annexin V‐FITC/PI double‐staining analysis. All data were representative of three independent experiments. Data represent means, and error bars are standard deviation. Two‐sided *t* test

The induction of apoptosis by MLN4924 prompted us to investigate the underlying mechanism. We measured the expression levels of classical pro‐apoptotic Bcl‐2 family members, including BH3‐only proteins Bid, Bim, Bik, Puma and Noxa as well as their downstream pro‐apoptotic proteins Bax and Bak in apoptotic HN13 and CAL27 cells. Among these proteins, pro‐apoptotic BH3‐only proteins Bik, Bim and Noxa were substantially up‐regulated in both protein and mRNA levels (Figure 4A,B), while protein levels of others barely changed (Figure 4A).

To evaluate the contributions of those pro‐apoptotic proteins in mediating MLN4924‐induced apoptosis, the expression levels of Bik, Bim and Noxa were firstly down‐regulated via siRNAs in MLN4924‐treated cells. We found that down‐regulation of Noxa demonstrated the most prominent rescue effect on MLN4924‐induced apoptosis (Figure 4C). In contrast, down‐regulation Bik and Bim only partially attenuated the MLN4924‐induced apoptosis (Figure S1B). These findings indicated a pivotal role of BH3‐only proteins, especially Noxa, in MLN4924‐induced apoptosis in HNSCC cells. To further verify the crucial role of Noxa in MLN4924‐induced apoptosis, Noxa was knocked out with CRISPR/Cas9 technology (Figure 4D). Compared with the control group, the percentage of Annexin V‐FITC/PI double‐positive cells induced by MLN4924 treatment were significantly decreased in Noxa knockout cells (Figure 4E).

### 3.5 CRL substrate c‐Myc was responsible for the transcriptional activation of Noxa

**Figure 5:**
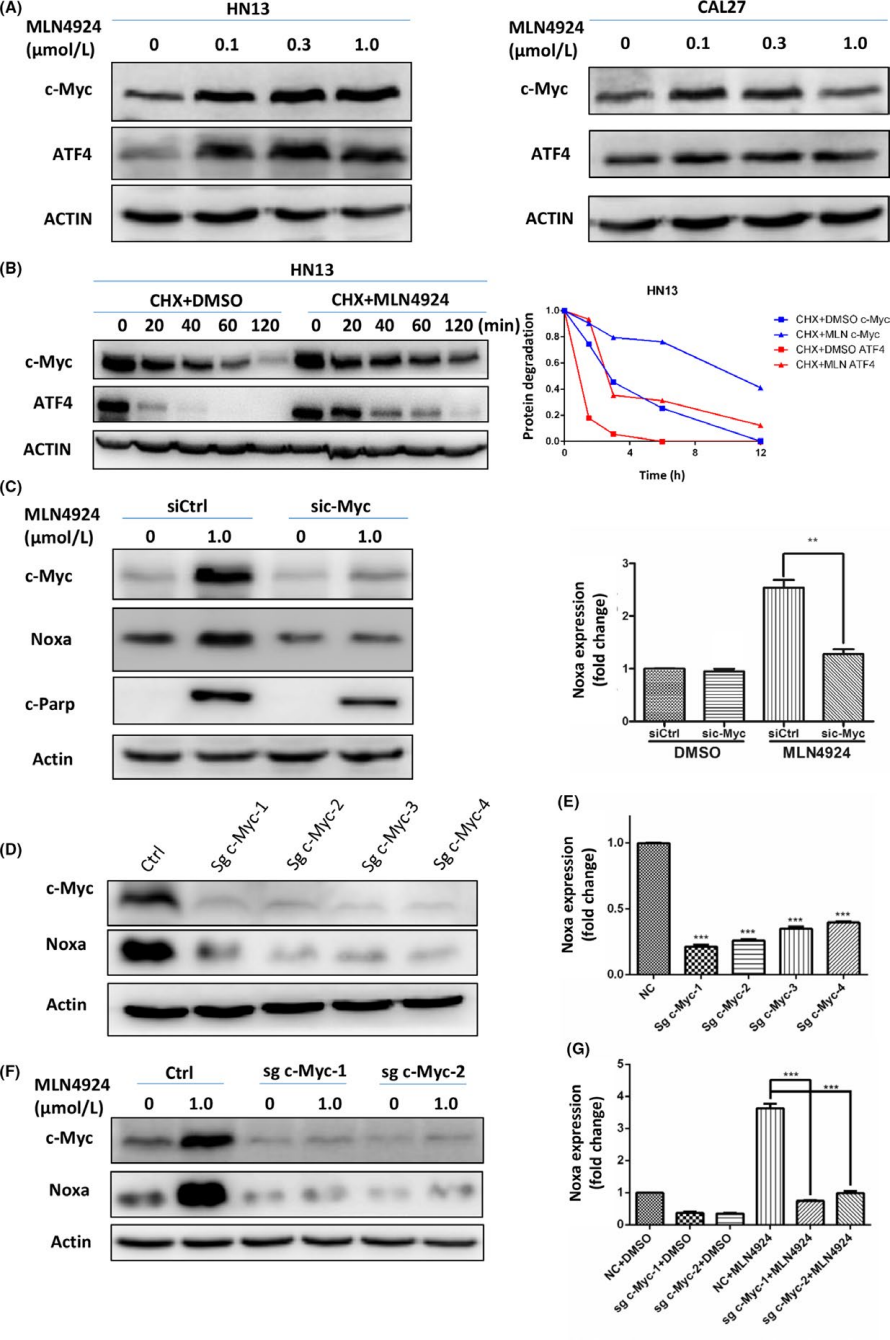
CRL substrate c‐Myc was responsible for the transcriptional activation of Noxa. (A) MLN4924 treatment led to the accumulation of c‐Myc and ATF4. Immunoblotting using antibodies against c‐Myc and ATF4 in HN13 and CAL27 cell lines was performed after 24 h of MLN4924 treatment at increasing concentrations (0.1, 0.3 and 1.0 μmol/L) vs DMSO. (B) MLN4924 prolonged the half‐lives of c‐Myc and ATF4. HN13 cells were pretreated with 0.3 μmol/L MLN4924 for 24 h and then treated with 1.0 μmol/L MLN4924 vs DMSO in combination with 50 μg/mL CHX for 0, 20, 40, 60 and 120 min and subjected to immunoblotting using antibodies against c‐Myc and ATF4 with actin as a loading control. The protein level was quantified by densitometric analysis. (C) C‐Myc accumulation led to Noxa transactivation. After HN13 cells transfected with control vs c‐Myc siRNA for 48 h, cultures were treated with DMSO or 1.0 μmol/L MLN4924 for another 48 h and collected for real‐time PCR for Noxa mRNA or subjected to immunoblotting using antibody against cleaved‐Parp (c‐Parp) and Noxa with actin as a loading control (**P <* *0.01, n = 3). (D‐G) C‐Myc knockout blocked Noxa transcriptional activation. (D) C‐Myc knockout efficiency was determined by Western blotting with actin as a loading control. Noxa protein and mRNA level in c‐Myc knockout cells were determined by Western blotting (D) and real‐time PCR (E, ***P < 0.001, n = 3). C‐Myc knockout cell lines were treated with DMSO or 1.0 μmol/L MLN4924 for 48 h and subjected to immunoblotting using antibody against Noxa with actin as a loading control (F) or collected for real‐time PCR for Noxa Mrna (G, ***P <* *0.001, n = 3). All data were representative of three independent experiments. Data represented means, and error bars were standard deviation. Two‐sided *t* test

Based on our above research, Noxa was transactivated upon neddylation inhibition and played a critical role in MLN4924‐induced apoptosis in HNSCC cells (Figure 4). We further identified the transcription factors responsible for Noxa induction upon neddylation inhibition. Previous studies demonstrated that activating transcription factor 4 (ATF4) and proto‐oncogene c‐Myc serve as substrates of CRL ubiquitin ligase and transcriptional activators of Noxa in a cell line‐dependent manner.^12^, ^19^, ^45^, ^46^ Therefore, ATF4 and c‐Myc were chosen for their CRL substrates identities as potential transcription factors for MLN4924‐induced Noxa activation in HNSCC.^19^, ^45^ As shown in Figure 5A, MLN4924 treatment induced the accumulation of both c‐Myc and ATF4. Furthermore, when blocking protein translation with CHX, MLN4924 significantly delayed the degradation of c‐Myc and ATF4 (Figure 5B). These findings collectively demonstrated that inhibition of neddylation‐CRL E3 ligase axis blocked the degradation of c‐Myc and ATF4, which finally lead to their accumulation.

To determine the specific transcriptional factor responsible for Noxa transactivation, the expression levels of c‐Myc and ATF4 were firstly down‐regulated via siRNA silencing in HNSCC cells. In MLN4924‐treated cells, down‐regulation of c‐Myc (Figure 5C) but not ATF4 (Figure S1C) significantly diminished the transactivation of Noxa. Consistently, knocking out of c‐Myc expression with CRISPR/Cas9 technology (Figure 5D) not only decreased Noxa transactivation (Figure 5E), but also abrogated MLN4924‐induced Noxa expression at both protein and mRNA levels completely (Figure 5F,G). Taken together, by blocking CRL‐mediated degradation of c‐Myc, MLN4924 activated the transcription of pro‐apoptotic protein Noxa and eventually induced cell apoptosis.

## 4 DISCUSSION

**Figure 6:**
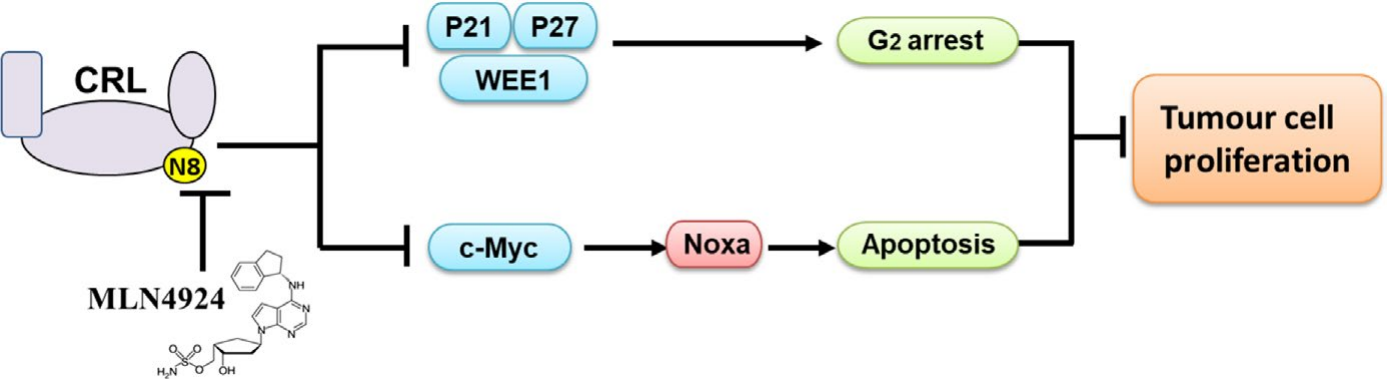
A working model of targeting neddylation pathway in HNSCC. N8 = NEDD8

Inhibition of neddylation pathway with NAE inhibitor MLN4924, a first‐in‐class anticancer agent, has been developed as a potential anticancer strategy for several human cancers.^9^, ^11^, ^13^, ^47^ In the present study, we demonstrated the inhibitory effects of MLN4924 on HNSCC malignant phenotypes such as cell proliferation and transwell migration and invasion ability. Mechanistically, the anticancer effects of MLN4924 on HNSCC cells were mediated by G_2_ phase cell cycle arrest and the ultimate induction of c‐Myc/Noxa axis‐dependent apoptosis (Figure 6). Our findings, in combination with previous reports demonstrating the preclinical and clinical effect of MLN4924 on HNSCC,^23^, ^29^, ^30^ provided the rationality for the use of neddylation inhibitors for the treatment of HNSCC in clinical trials.

Induction of cell cycle arrest in cancer cells upon neddylation inhibition is an initial response, frequently proceeding apoptosis.^48^, ^49^ Previous studies showed that MLN4924 could induce S phase and G_1_ phase cell cycle arrest in some cancer cells.^14^, ^50^, ^51^ The later studies from our and other's groups demonstrated that MLN4924 mainly trigger cell cycle arrest at the G_2_ phase in many types of cancer cells.^9^, ^10^, ^11^, ^33^, ^52^, ^53^, ^54^, ^55^, ^56^, ^57^, ^58^, ^59^, ^60^, ^61^ Consistently, genetic inhibition of CRLs by RBX1/ROC1 knockdown induces G_2_ phase arrest in cancer cells as well.^32^, ^62^, ^63^, ^64^ In this study, we demonstrated that neddylation inhibition by MLN4924 triggered G_2_ phase arrest in HNSCC cells by inducing the accumulation of CRL substrates P21, P27 and WEE1. These findings demonstrate that neddylation inhibition induces cell cycle arrest at different phases in a cell type‐dependent manner.

During addressing the mechanisms for MLN4924‐induced intrinsic apoptosis in HNSCC cells, we defined that BH3‐only protein Noxa plays a crucial role in this process. Chen et al^12^ recently found that in oesophageal squamous cell carcinoma (ESCC) cells, MLN4924‐induced Noxa activation was dependent on ATF4, a well‐known CRL substrate, and down‐regulation of ATF4 completely blocked the induction of Noxa. In contrast, Knorr et al^19^ reported that c‐Myc, another CRL substrate, transactivated Noxa and triggered intrinsic apoptosis upon neddylation inhibition in acute myelogenous leukaemia cells. Interestingly, we found in this study that both c‐Myc and ATF4 accumulated upon MLN4924 treatment in HNSCC cell lines. However, the knockdown of c‐Myc but not ATF4 significantly diminished the transactivation of Noxa and attenuated the induction of apoptosis in HNSCC cells. These findings collectively indicated that MlN4924‐induced Noxa transcriptional activation is cell line‐dependent. Further elucidation of the intrinsic mechanisms of cell line‐dependent Noxa transactivation by c‐Myc or ATF4 is warranted.

Transwell cell migration and invasion assay demonstrated MLN4924 treatment dramatically reduced HN13 and Cal27 cell migratory and invasive properties. Zhu et al.^65^ have demonstrated MLN4924 impeded the transwell cell migration and invasion assay in line with decreased epithelial‐to‐mesenchymal transition (EMT) markers such as fibronectin and N‐cadherin in breast cancer cell line MDA‐MB‐231. However, neither our nor Zhu's research work involved in vivo data on mice models, which means the inhibitory effect of MLN4924 on distant metastatic spread remaining uncertain. Certain orthotopic and metastasis model of HNSCC would have been applied to observe the inhibitory effect both on primary and metastatic tumours.^66^, ^67^, ^68^ Additionally, given that treatment of MLN4924 induced G2‐M phase cell cycle arrest in 24 h (Figure 2A), impact of MLN4924 on cell proliferation also contributed to the diminution of the cell number observed in transwell assays.

Taken together, our study revealed the mechanisms underlying MLN4924‐induced G_2_ phase cell cycle arrest and apoptosis in HNSCC cells, which provided scientific basis for further clinical evaluation of MLN4924 for the treatment of HNSCC.

## AUTHOR CONTRIBUTIONS

LJJ and YMZ designed and supervised the project. WJZ carried out the experiments and drafted the manuscript, LJJ finalized the manuscript. LSJ and JHY synthesized and provided the small molecular inhibitor MLN4924. YPL, LHL and XFW designed the real‐time qPCR primer and performed real‐time qPCR assay. ZY performed the TCGA analysis. MSZ, QZ and XKL collected the HNSCC clinical samples. SYS provided HNSCC cell lines. CSD, XJL, JHK and HZ performed statistical analysis and helped to draft the manuscript. All authors read and approved the final manuscript.
