# Supplementary figures and images for: The Nedd8‐activating enzyme inhibitor MLN4924 (TAK‐924/Pevonedistat) induces apoptosis via c‐Myc‐Noxa axis in head and neck squamous cell carcinoma
Source: Cell Prolif. 2018 Oct 19;52(2):e12536. doi: 10.1111/cpr.12536 (PMC6496207; doi:10.1111/cpr.12536)

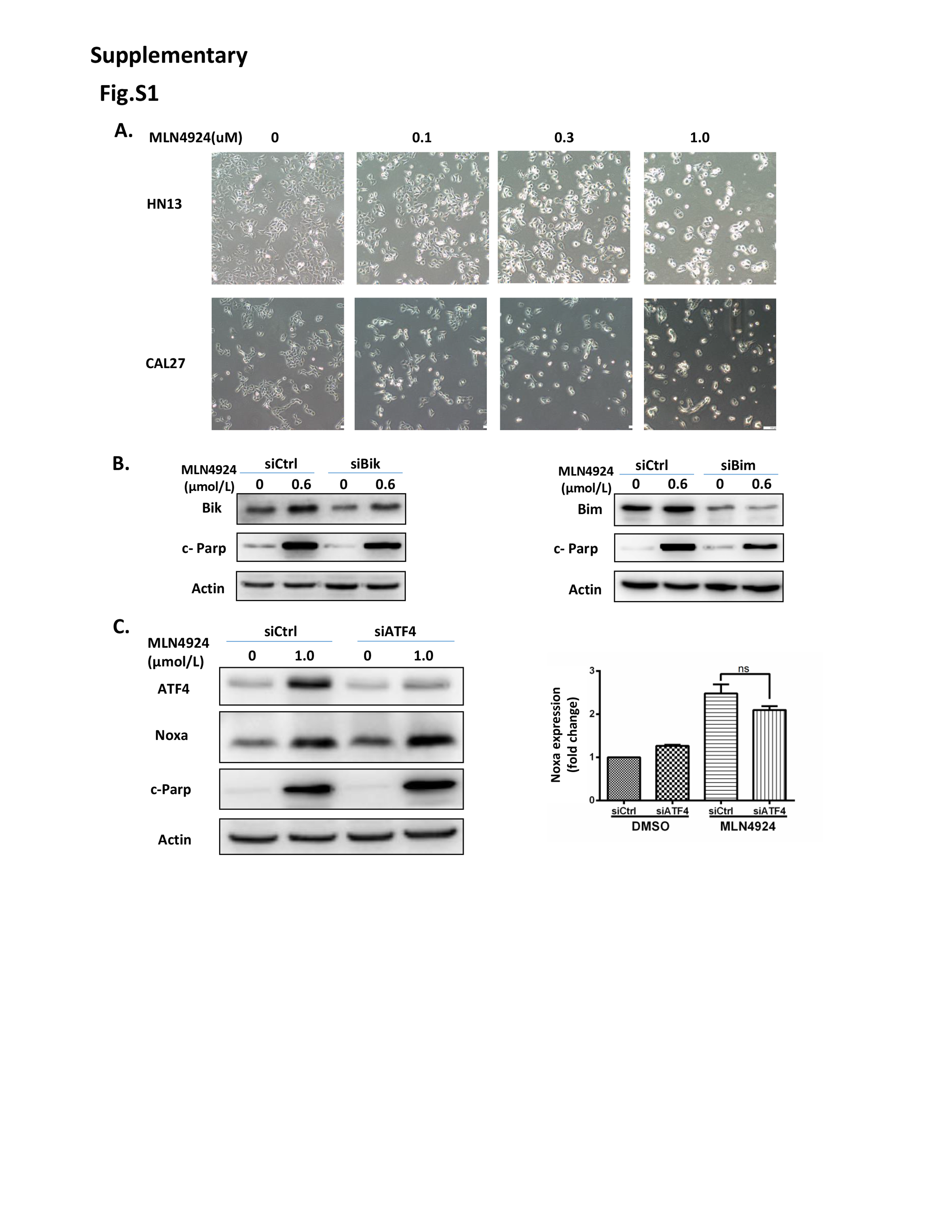

Supplement: Supplementary file 1 [file CPR-52-e12536-s001.tif]
