# Supplementary material for: The Nedd8‐activating enzyme inhibitor MLN4924 (TAK‐924/Pevonedistat) induces apoptosis via c‐Myc‐Noxa axis in head and neck squamous cell carcinoma
Source: Cell Prolif. 2018 Oct 19;52(2):e12536. doi: 10.1111/cpr.12536 (PMC6496207; doi:10.1111/cpr.12536)
Supplement: Supplementary file 2 [file CPR-52-e12536-s002.docx]

**Table S1 - List of SgRNA oligos sequences in this study**

| Name | Sequence(5'-3') |
| --- | --- |
| Noxa-1-F | CACCGTCGAGTGTGCTACTCAACTC |
| Noxa-1-R | AAACGAGTTGAGTAGCACACTCGAC |
| Noxa-2-F | CACCGACGCTCAACCGAGCCCCGCG |
| Noxa-2-R | AAACCGCGGGGCTCGGTTGAGCGTC |
| Noxa-3-F | CACCGTTCTTGCGCGCCTTCTTCCC |
| Noxa-3-R | AAACGGGAAGAAGGCGCGCAAGAAC |
| c-Myc-1-F | CACCGAACGTTGAGGGGCATCGTCG |
| c-Myc-1-R | AAACCGACGATGCCCCTCAACGTTC |
| c-Myc-2-F | CACCGGCCGTATTTCTACTGCGACG |
| c-Myc-2-R | AAACCGTCGCAGTAGAAATACGGCC |
| c-Myc-3-F | CACCGTGCGTAGTTGTGCTGATGTG |
| c-Myc-3-R | AAACCACATCAGCACAACTACGCAC |
| c-Myc-4-F | CACCGACAACGTCTTGGAGCGCCAG |
| c-Myc-4-R | AAACCTGGCGCTCCAAGACGTTGTC |
